# An ultra-high gain and efficient amplifier based on Raman amplification in plasma

**DOI:** 10.1038/s41598-017-01783-4

**Published:** 2017-05-25

**Authors:** G. Vieux, S. Cipiccia, D. W. Grant, N. Lemos, P. Grant, C. Ciocarlan, B. Ersfeld, M. S. Hur, P. Lepipas, G. G. Manahan, G. Raj, D. Reboredo Gil, A. Subiel, G. H. Welsh, S. M. Wiggins, S. R. Yoffe, J. P. Farmer, C. Aniculaesei, E. Brunetti, X. Yang, R. Heathcote, G. Nersisyan, C. L. S. Lewis, A. Pukhov, J. M. Dias, D. A. Jaroszynski

**Affiliations:** 1 Department of Physics, Scottish Universities Physics Alliance and University of Strathclyde, Department of Physics, Glasgow, G4 0NG United Kingdom; 2 Institute of Physics of the ASCR, ELI-Beamlines, Na Slovance 2, 182 21 Prague, Czech Republic; 3 GoLP/Instituto de Plasmas e Fusão Nuclear, Instituto Superior Técnico, Universidade de Lisboa, Lisbon, Portugal; 4 IFIN-HH, National Institute for Physics and Nuclear Engineering, Bucharest, Romania; 5 UNIST, Banyeon-ri 100. Ulju-gun, Ulsan, 689-798 South Korea; 6 Theoretische Physik I, Heinrich Heine Universität, 40225 Düsseldorf, Germany; 7 Central Laser Facility, Rutherford Appleton Laboratory, Didcot, OX11 0QX United Kingdom; 8 Centre for Plasma Physics, School of Mathematics and Physics, Queens University Belfast, Belfast, BT7 1NN United Kingdom; 9 Diamond Light Source, Harwell Science and Innovation Campus, Fermi Ave, Didcot, OX11 0DE UK; 10 Lawrence Livermore National laboratory, NIF and photon Sciences, 7000, East avenue, Livermore, CA 94550 USA; 11 Centre de Physique Théorique, École Polytechnique, 91128 Palaiseau cedex, France; 12 Medical Radiation Science, National Physical Laboratory, Medical Radiation Science, Hampton Road, Teddington, Middlesex TW11 0LW UK; 13 Center for Relativistic Laser Science, Institute for Basic Science, Gwangju, 61005 Republic of Korea; 14 Department of Physics, Capital Normal University, Key Lab of Terahertz Optoelectronics, Ministry of Education, and Beijing Advanced Innovation Center for Imaging Technology, Beijing, 100048 China

## Abstract

Raman amplification arising from the excitation of a density echelon in plasma could lead to amplifiers that significantly exceed current power limits of conventional laser media. Here we show that 1–100 J pump pulses can amplify picojoule seed pulses to nearly joule level. The extremely high gain also leads to significant amplification of backscattered radiation from “noise”, arising from stochastic plasma fluctuations that competes with externally injected seed pulses, which are amplified to similar levels at the highest pump energies. The pump energy is scattered into the seed at an oblique angle with 14 J sr^−1^, and net gains of more than eight orders of magnitude. The maximum gain coefficient, of 180 cm^−1^, exceeds high-power solid-state amplifying media by orders of magnitude. The observation of a minimum of 640 J sr^−1^ directly backscattered from noise, corresponding to ≈10% of the pump energy in the observation solid angle, implies potential overall efficiencies greater than 10%.

## Introduction

Stimulated Raman scattering in plasma has been extensively investigated over the last three decades. Studies were initially motivated by the need to understand parametric instabilities occurring in laser-driven fusion experiments (e.g. see ref. 1). However, more recently, stimulated Raman backscattering (SRBS) has been studied both experimentally^2–14^ and theoretically^15–38^, as potential compact plasma-based laser amplifiers/compressors that could pave the way to achieving exawatt laser powers.

Stimulated Raman scattering is a three-wave interaction where an electromagnetic (EM) wave of frequency *ω*_0_ and wave vector **k**_**0**_ transfers energy to a lower frequency EM wave (*ω*_1_, **k**_1_) through resonant excitation of a plasma wave (${\omega }_{p}=\sqrt{{n}_{e}{e}^{2}/{\varepsilon }_{0}{m}_{e}}$, **k**_**p**_), which occurs when ${\omega }_{0}\approx {\omega }_{1}+{\omega }_{p}$ and ${{\bf{k}}}_{{\bf{0}}}\approx {{\bf{k}}}_{{\bf{1}}}+{{\bf{k}}}_{{\bf{p}}}$ ^39^. *n*_*e*_ is the electron plasma density, *ε*_0_ the permittivity of vacuum, and −*e* and *m*_*e*_ are the electron charge and mass, respectively. While the linear regime of stimulated Raman backscattering is considered unsuitable for amplification of ultra-short laser pulses because of pulse lengthening^16, 40^, the nonlinear^8, 9, 41^, autoresonance^30^ and wavebreaking regimes^26, 36–38^ are potentially efficient ways of obtaining ultra-short duration and ultra-intense laser pulses. However, the highest efficiency measured to date for double-pass amplification is 6.4%, for amplification of a 16 μJ pulse to a 5.6 mJ pulse^9^. Unfortunately, this is almost an order of magnitude lower than what simulations predict, even when taking Raman forward scattering, filamentation and wavebreaking into account^22, 26^. The discrepancies between experiments and theoretical predictions have driven the search for a better understanding of what limits their efficiency.

Amplification is governed by the relative phase and amplitude of the resonantly excited plasma echelon. Saturation of the plasma wave amplitude arises from several processes, e.g. detuning caused by Landau damping/trapped electrons^18^, optical frequency chirps^12, 14, 19, 42^, group velocity dispersion^29^, plasma mode coupling^43^ and the simultaneous excitation of Raman, Brillouin and electron-acoustic scattering^13^. To interpret experiments it is necessary to incorporate these complex processes into theoretical models. Some are easily included in particle-in-cell (PIC) simulations, but inverse bremsstrahlung (IB), which is responsible for plasma heating and Bohm-Gross shifts, is difficult to model or computationally inefficient and therefore is often not included.

In this paper, we investigate Raman amplification of extremely low intensity seed pulses with pump pulse energies up to 100 J. The large range of pump intensities, up to 10^17^ W cm^−2^ available, has enabled a detailed study of amplification and competing Raman amplification from noise to be carried out. The feasibility of amplification in a counter-propagating geometry is evaluated by measuring the angular energy density of Raman amplification from noise both in the direct backscattered direction and at an oblique angle. This demonstrates efficiencies in excess of 10%. While simulations are not yet fully able to model realistic systems because of their susceptibility to numerical instabilities, dispersion and enhanced statistical noise, qualitative observations compare well with the experimental results. In the Supplementary Information we also discuss the role of wavebreaking that may occur in the period prior to the arrival of the seed pulse.

## Experimental setup

The results presented here have been obtained during two experimental campaigns performed at the Vulcan laser facility at the Rutherford Appleton Laboratory (RAL), which are described below. Further details can be found in Supplementary Information.

The *λ*_0_ = 1.053 μm pump pulse has on-target energies of up to 100 J and a duration of 10 ps (FWHM). This is focused into a 3-mm-long hydrogen gas jet by a *f*/21 lens, giving a ~50 μm focal waist radius (radius at 1/*e* ^2^ in intensity with 25–30% of the energy within the beam waist). Amplification is studied for pump *peak* intensities of up to 10^17^ Wcm^−2^ (calculated from the focal spot image), which has enabled observation of non-linearities and competing effects at high power densities.

The seed is produced by down-shifting a 1-ps duration, 1.053 μm laser pulse to 1.138 μm (close to the theoretical value of 1.147 μm), in a KGW Raman crystal^44^. It is focused into the gas jet using a *f*/40 plano-convex lens. However, poor beam quality due to non-linearities in KGW enlarge the focal waist to ≈1.5 mm, which results in the pump beam being fully enclosed by the seed, i.e. the seed cross-section is much larger than the pump cross-section. As a result, only a few100s of pJ of the ≈130 nJ seed pulse interacts with the pump within the ionized gas volume. The seed spectrum has a Lorentzian profile with a full width at half maximum (FWHM) of 15 nm.

The nearly counter-propagating pump and seed form an angle of 175° with respect to each other to avoid feedback into the laser chain. Therefore, the interaction lengths for different parts of the seed are constrained to $\sim 2{w}_{0}/\,\sin \,5^\circ \approx 1.1\,{\rm{mm}}$, where *w*_0_ is the pump waist.

To obtain resonance between pump and seed, a plasma density of 6–7 × 10^18^ cm^−3^ is required, which is achieved by focusing 1.25 mm above the gas jet nozzle with a backing pressure of 12 bars. The ionization threshold of hydrogen allows for intensities from 10^14^ W cm^−2^ upwards.

## Experimental results

Here we compare the Raman signal characteristics of both seeded Raman scattering (S-RS) and amplified noise Raman scattering (N-RS) as a function of pump intensity. To determine the extent of N-RS we compare the energy scattered in the oblique (175°) direction with that backscattered in the direct counter-propagating direction. The focused pump and seed beams define a solid angle of 1.8 × 10^−3^ sr and 5 × 10^−4^ sr, respectively, while the detection solid angles are 7.8 × 10^−3^ sr on-axis and 5 × 10^−3^ sr off-axis.

### Energy measurements

**Figure 1:**
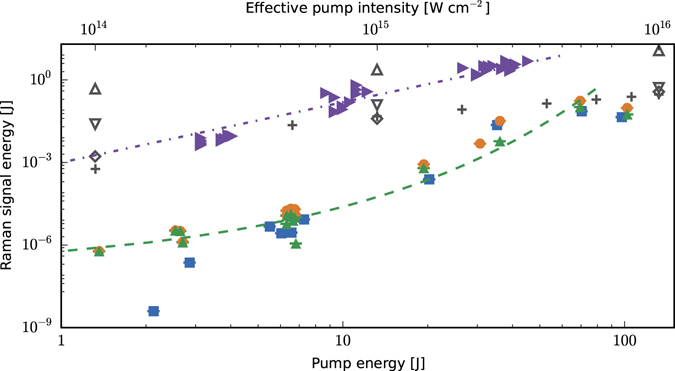
Measured and numerically calculated Raman signal energies. Experimental (numerical) results are shown by filled (empty, gray) symbols. (Blue) square: N-RS; (orange) circle: S-RS + N-RS; (green) up-pointing triangle: S-RS with the (green) dashed line showing an exponential fit with respect to the square root of the pump energy. For comparison, results from simulations are shown. Cross: results from Leap. down-pointing triangle: OSIRIS simulations; Diamond: cplPIC simulations. The effective pump intensity is an estimated value used to enable comparison between experimental and simulation results. See Methods for details. Measured Raman signal energies backscattered along the pump axis are presented (right-pointing, purple triangle) in addition to results from OSIRIS simulations for a 2.6 mm long plasma (up-pointing triangle). (Purple) dash-dot line: power law fit to on-axis scattering data with exponent 2.2. Note that the horizontal lines across the symbols are error bars.

To determine the energy gain and efficiency in the oblique direction, the energy of the Raman signal has been measured as a function of pump energy both with and without the presence of the external seed, as shown in Fig. 1, where we identify the two respective measurements by the labels (*S-RS* + *N-RS*) and (*N-RS*). The Raman signal energy is observed to increase from 4 nJ(N-RS)/1 μJ(S-RS + N-RS) to 70 mJ/170 mJ, respectively, as the nominal pump energy is increased from 1 to 70 J. At the highest pump energies the seed is amplified by nearly nine orders of magnitude. Because the seed interaction length is 1.1 mm at 175°, the observed maximum gain in 5 × 10^−3^ sr sets a lower limit to the gain coefficient of 180 cm^−1^, which is two orders of magnitude larger than conventional high power solid state amplifying media.

For pump energies of ~1 J, the amplified seed pulse is a factor of 250 larger than the noise signal, which is sufficient for pJ seed signals to be amplified above the noise. At higher pump energies, this ratio drops to 2.5 (at 70 J), which shows that N-RS can reach significant levels, comparable with S-RS. Because N-RS depends on both the particle density and temperature at any time, and is integrated in time convectively, the seed sees a spatio-temporal evolving gain medium, as we show later, which accounts for the variation of the gain. Above 70 J the Raman signal appears to drop, but this should be interpreted cautiously because the laser beam quality degrades rapidly at higher energies. Furthermore, significant N-RS energy is scattered directly backwards with an energy angular density of up to 50 times that of obliquely scattered radiation, as is discussed later.

To distinguish between the energy gained by the seed, S-RS, and that resulting from N-RS, the two sets of measurements are subtracted. We find that in the range of 600 nJ to 100 mJ the amplified seed energy, *ε*_1_, approximately fits an exponential dependence, ${\varepsilon }_{1}({\varepsilon }_{0})\propto \exp ({c}_{1}\sqrt{{\varepsilon }_{0}})$, on the pump energy, *ε*_0_, up to a pump energy of 70 J, where *c*_1_ is a fitting parameter. However, the growth rate is much lower than predicted theoretically for the linear regime. We observe that the total efficiency measured in the oblique (175°) direction, within 5 × 10^−3^ sr, is less than 0.5%, when only considering the energy of the amplified seed, and around 1% for the total signal. As we show below, the total fraction of the pump that is directly backscattered can be significantly larger, which suggests that much higher efficiencies, in excess of 10% should be possible for properly angular matched collinear Raman amplification geometries.

### Transverse profile of the Raman signal

**Figure 2:**
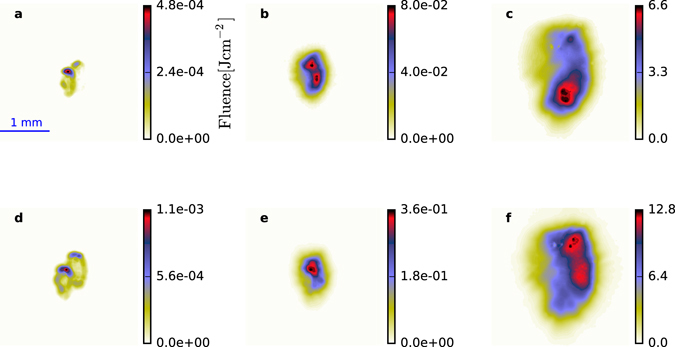
Transverse profile of the Raman signal. Recorded beam profiles for three different nominal pump energies: 3 J (**a**,**d**), 20 J (**b**,**e**) and 70 J (**c**,**f**). (**a–c**) are obtained from amplification from noise, while (**d–f**) are recorded with external seed injection. Note the very different fluence scales.

Measurement of the transverse profiles of the obliquely backscattered Raman radiation provides information on the beam quality and peak fluence. A selection of six typical shots for three different nominal pump energies, 3, 20 and 70 J, are presented in Fig. 2. Figures 2a–c illustrate N-RS shots, while Fig. 2d–f represent S-RS + N-RS shots. At comparable pump energies, S-RS + N-RS and N-RS profiles are similar. The transverse energy distribution is inhomogeneous and the scattered radiation profile seems mainly to be determined by the (poor) optical characteristics of the pump beam. Increasing the pump energy results in larger volumes of ionized gas, i.e., larger scattering volumes, which is accentuated by the non-uniformity of the pump transverse profile. As the pump energy is varied between 1 and 70 J, the cross-section area of the backscattered light grows by an order of magnitude. The largest fluence measured from the radiation profiles is 12.8 J cm^−2^ for injection of the external seed. This is still two orders of magnitude less than predicted theoretically for an efficient Raman amplifier^33^. However, it demonstrates the potential of the medium for amplifying seed pulses with initial energies as low as a few 100s of pJ for non-optimal pump beams.

### Spectral analysis

**Figure 3:**
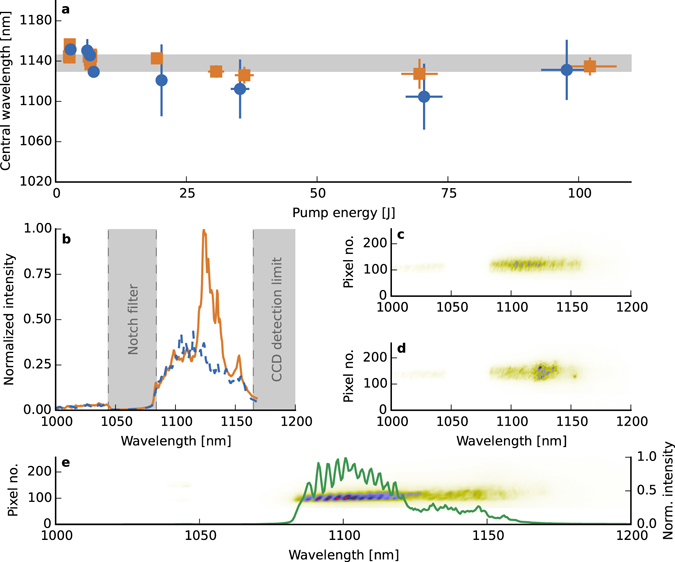
Spectra analysis. (**a**) Comparison between the central wavelengths of (blue circle) N-RS spectra and (orange square) S-RS + N-RS spectra. The error bars in the vertical axis represent the measured bandwidth at FWHM. The grey area illustrates the initial seed features. (**b**) Examples of spectra for a nominal pump energy of 35 J: N-RS spectrum shown in dashed (blue) line, S-RS + N-RS spectrum presented in solid (orange) line. (**c**) and (**d**) spectral images of Raman amplification without and with seed, respectively. The spectra correspond to the ones presented in (**b**). (**e**) example of a spectrum showing strong modulations, obtained for a 6 J pump beam only.

The measured signal spectra, shown in Fig. 3, are typical spectra obtained for a pump energy of ~35 J, which reveal several interesting features. The N-RS spectra are broad and roughly Gaussian in profile, while for S-RS a narrow peak is superposed on the N-RS feature (Fig. 3b). The central wavelengths and the spectral widths are shown in Fig. 3a, where it is observed that the spectral width of the amplified seed varies between 10 and 20 nm, which is close to the original KGW spectral width. However, an increase in the N-RS signal is accompanied by a rapid broadening of the spectral bandwidth, from 10 nm at low pump energies to 60 nm at 20 J and above. This is consistent with noise amplification^45^, where the bandwidth of the N-RS spectra is determined by the linear gain bandwidth. For joule-level (1–2 J) pump energies, the central wavelength of the spectra remains around 1.15 μm for both sets of measurements. This value is very close to the seed central wavelength, which confirms that the plasma is resonant. Above several joules, the central wavelength of the N-RS spectra is strongly blue-shifted by up to 50 nm, which is consistent with trapping of electrons in the plasma wave, which leads to an effective local reduction in the plasma density^14, 46^. This is confirmed by a less pronounced blue shift in S-RS spectra of approximately 20 nm, which is within the initial bandwidth of the seed and shows that the lower intensity blue parts of the spectrum are preferentially amplified when the pump energy is low and Raman gain bandwidth is narrow. The increase in the gain bandwidth at very high pump intensities also reduces these bandwidth effects, as is evident in Fig. 3a. Measurement of seed spectra show that it maintains its initial spectral characteristics, particularly at low pump energies, which demonstrates the fidelity of the amplifier, with some evidence of nonlinearities at higher intensities. However, it should be noted that spectral features at wavelengths beyond ~1.165 μm, the CCD’s sensitivity range limit, cannot be observed, if present.

### Stimulated Raman backscattering from noise

Our studies show that N-RS becomes significant, and even dominant, as the pump intensity is increased, even at large angles. At pump intensities of around 10^14^ W cm^−2^ (*a*_0_ ≈ 0.01), the level of amplified noise is more than two orders of magnitude below that of the amplified seed. However, the ratio of S-RS to N-RS decreases rapidly as the pump intensity increases because the initial noise signal is proportional to the pump intensity.

**Figure 4:**
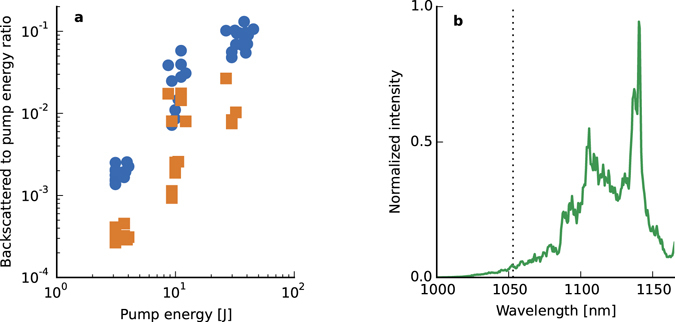
Measurements of backscattered energy. (**a**) Ratio of (blue circle) backscattered Raman and (orange square) elastic/Brillouin scattering energy to initial pump energy. Up to 10% of the pump energy is converted to SRBS. Elastic/Brillouin scattering accounts for one order of magnitude less. (**b**) Example of a corrected spectrum obtained for a pump energy of 38 J. An estimated backscattered energy of 5 J is measured.

The total N-RS energy in the direct-backscattered direction can become significant because the interaction length and gain are maximised in this direction, for a Fresnel number (of the central spot) *F* ≈ 1. To investigate N-RS, light transmitted through the final pump mirror (before the gas jet) is measured using a calorimeter, for energy measurements, and an imaging spectrometer, for spectral measurements. The on-axis collection solid angle is 7.8 × 10^−3^ sr, which is 1.6 times larger than for off-axis measurements. Figure 4 shows the ratio of the Stokes-shifted (Raman) energy compared with (near-)unshifted (elastic/Brillouin) scattering, for pump energies ranging from 1 to 50 J. At 50 J approximately 5 J (10%) of the pump energy is converted to backscattered radiation, giving an angular energy density of 640 J sr^−1^, calculated by assuming the scattered radiation fills the collection solid angle. This underestimates the efficiency as some pump energy is scattered outside the measurement solid angle. The backscattered energy is almost fifty times larger for 50 J than what is collected off-axis for 70 J, where the maximum angular energy density is 14 J sr^−1^ (70 mJ). These measurements are consistent with N-RS strongly dominating for our interaction geometry, *F* ≈ 1, even at low pump intensities. At higher energies noise backscattering is dominant and can exceed 10%. Elastic scattering is at least one order of magnitude lower. To compare the energy measurements of N-RS in the direct-backscattered direction with off-axis scattering we have also included this data in Fig. 1, notwithstanding the different collection angles. The scaling law follows a dependence on the pump energy of ${\varepsilon }_{0}^{2.2}$, which may indicate amplification in the superradiant regime with the noise source being proportional to the pump laser power, giving an overall scaling of ${\varepsilon }_{0}^{2.5}$.

## Discussion and numerical simulations

In the experiments we observe that S-RS and N-RS exhibit exponential gain with a growth factor that is much smaller than expected from linear theory, for a fixed interaction length. We also observe N-RS to scale as ${\varepsilon }_{0}^{2.2}$ on axis with more than 10% of the energy backscattered at 50 J pump energies. To interpret these results we consider two main hypotheses. The first hypothesis is that the interaction length depends on the pump intensity: an increase in pump intensity leads to wave breaking, and/or noise driven superradiance, which shortens the length of relatively undisturbed plasma where the seed can be efficiently amplified. In the second hypothesis, the growth factor is reduced due to damping of the plasma wave amplitude.

To gain a deeper understanding of the observed amplification we have undertaken numerical simulations using OSIRIS^47^, cplPIC (based on ref. 48) and Leap^49^. The input parameters are chosen to match the experimental ones (see the Methods and Supplementary Information sections for details).

For near-perfect matching conditions, the three codes produce comparable results but fail to quantitatively reproduce the measured energy trend. Raman signal energies from the simulations are shown in Fig. 1 to enable direct comparison with the experimental results. While agreement is obtained at high energy, the gain in the simulation is 10^7^ for the lowest pump intensity (10^14^ W cm^−2^), whereas 10^4^ is measured experimentally. The N-RS energy is strongly enhanced by the reduced number of macro-particles, ${n}_{m}$, because the noise signal scales as $1/\sqrt{{n}_{m}}$. In the simulations the strong noise-driven source also depends on the pump energy. Rapid amplification quickly leads to particle trapping and breaking of the plasma wave, which masks amplification of the seed (the first hypothesis). However, the measured scattered energy is also consistently low for low pump energy. This implies that a different mechanism reduces the scattering efficiency, most likely collisional and/or Landau damping, whereas wavebreaking is expected not to be important at low pump energies.

To test our second hypothesis on damping of the plasma wave due to Landau damping and thermal effects, we have carried out cold plasma simulations using Leap with fixed damping factors. Initial results show that damping delays the onset of saturation, giving exponential growth for lower pump intensities (See Supplementary Information).

Simulations in warm plasma using cplPIC highlight the importance of nonlinear effects and show that filamentation can develop even at low pump intensities, and Raman side scattering may be important for moderate pump intensities, consistent with our measurements that show large spectral broadening (details in the Supplementary information).

**Figure 5:**
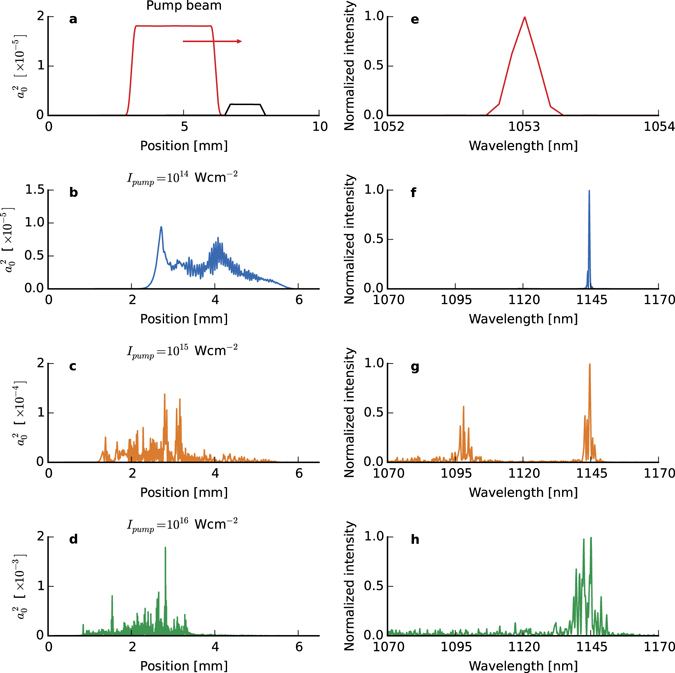
1-dimensional simulation results. (**a**) (red) Initial pump envelope for an intensity of 1 × 10^14^ W cm^−2^ and (**e**) corresponding spectrum. In (**a**) is also shown (black) the position of the plasma. (**b–d)** backscattered Raman signal for 3 different pump intensities (*a*_0_): 10^14^ (0.01), 10^15^ (0.03) and 10^16^ cm^−2^ (0.1), respectively. (**f–h**), Corresponding spectra.

Finally, 1D simulations have been carried out using OSIRIS with a fixed frame (i.e. no moving window) to qualitatively evaluate the amount of N-RS energy back scattered, which also clearly demonstrates an enhancement due to the reduced number of particles used in the simulations. Figure 5 shows the evolution of N-RS from a long backscattered pulse at low pump intensity, as expected from the linear theory (Fig. 5b), to a spiky structure, accompanied by spectral modulations (Fig. 5d,h) at higher pump intensity when strong wavebreaking and particle trapping occurs. The spiky modulations have a similar periodicity to those observed in experiments, as shown in Fig. 3e. A strong spectral component that is up-shifted by ${\omega }_{p}\mathrm{/2}$, as illustrated in Fig. 5g, is observed for pump intensities of 10^15^ W cm^−2^. This is evidence of a limit cycle^50^ that leads to period doubling followed by a cascade, which are similar to that occurring in saturated free-electron lasers and damped, driven nonlinear oscillators, where sidebands, chaotic behaviour and broad spectral bandwidths^51^ are observed even at modest intensities. This suggests that the strength of the ponderomotive potential is of the same order of magnitude as the electrostatic potential, and therefore the amplifier operates in an intermediate regime between the Raman and superradiant or Compton regimes. A realistic estimate for the bounce frequency, *ω*_*B*_, gives ${\omega }_{B}\ge {\omega }_{p}\mathrm{/2}$ for a pump intensity larger than 10^15^ W cm^−2^. In this case, superradiance occurs in segmented regions of the plasma, each arising from different noise spikes, and is dominated by synchrotron behaviour of electrons in the potential, which gives rise to sidebands that produce a modulated spectrum.

Both experiment and simulations suggest that a pump intensity below 1 × 10^15^ W cm^−2^ is preferred for amplification as N-RS is significantly reduced, which ensures that the plasma medium is not significantly disrupted. In addition, a lower intensity would limit the amount of Raman side scattering and filamentation. The Supplementary Information section presents further 1-D simulations to illustrate the deleterious effect of wavebreaking/particle trapping on the amplification even for moderate intensity pumps.

## Conclusions

In conclusion, a large Raman backscattered energy of up to 170 mJ has been measured for a monochromatic 70 J pump pulse incident at an angle of 175°. Injected pJ seed pulses are observed to grow by eight orders of magnitude, corresponding to a gain coefficient of 180 cm^−1^. However, 70 mJ of the backscattered pump is attributed to amplification of noise, which is proportional to the pump intensity and inversely proportional to the square root of the plasma density. Operating with a pump intensity below 10^15^ W cm^−2^ is desirable because: (i) noise amplification is suppressed; (ii) Raman side scattering is reduced; and (iii) the onset of noise amplification and wavebreaking prior to the arrival of the seed, leading to gain reduction or suppression, is delayed.

Measurements of N-RS along the pump axis indicates that more than 10% of the pump energy can be backscattered. This clearly shows the potential of the amplifying medium for high efficiency amplification. Finally, to avoid the deleterious effects of N-RS, tailoring of the plasma density and/or a frequency chirped pump may be required^16^. Otherwise, short laser pulse amplification starting in the Raman regime is limited by chaotic non-linear behaviour for a beatwave above a certain amplitude. We also observe that the seed profile is dominated by the pump profile, which it mimics.

## Methods

### Diagnostics

A 3″ wedge pick-off is used to collect 1% of the light along the seed axis after interaction, which is then transported to a diagnostics table. A calorimeter is set up behind the wedge to directly measure the seed energy. The diagnostics system consists of a CCD camera, which images the seed beam at the gas jet, and an imaging spectrometer. In addition, neutral density filters attenuate the signal energy and notch filters are used to attenuate radiation at 1.053 μm. The diagnostics setup for the pump includes a ×10 magnification imaging system and a CCD camera, for beam alignment and observation, and a linear array spectrometer and calorimeter.

### Experimental data analysis

The set of data presented represents the best single shot measurements obtained. The spectral responses of all neutral density filters have been measured to correct/calibrate the recorded spectra. Theoretical values for the CCD camera response, the spectrometer grating and mirrors are also used for the calibrations. When the Raman signal energy is below the threshold of the calorimeter, it is deduced from the number of counts on the imaging camera, which is energy calibrated. Furthermore, corrections/calibration of the spectra and energy measurements for the Raman signal directly backscattered along the pump axis have been obtained from a reliable theoretical transmission curve of the coating of the mirror through which light leakage is collected. Finally, the effective pump intensities used in Fig. 1 are calculated by mapping the intensity profile of the pump focal spot images in vacuum recorded for different energies. The effective intensity is then evaluated by averaging the 1% highest values from the image. A linear fit is then performed between energy and intensity.

### 2-dimensional PIC simulations

cplPIC is an averaged envelope code coupled to a standard PIC particle solver^48^. The vector potential for the laser is separated into a complex envelope and phase. The wave equation for the envelope is averaged over the beat wavelength for the two colliding pulses and solved numerically to update the envelope. Second-order derivatives are retained, relaxing the slowly-varying approximation. The response of the plasma to the laser pulses is evaluated from the ponderomotive force at the position of the particles. Charge is deposited on the grid and used to calculate the electric and magnetic fields caused by the plasma, which are evaluated and updated using Maxwell’s equations. The fields are then interpolated to the positions of the particles, which are then pushed using the Boris method. The envelope for the vector potential is reduced to a single (complex) component, which is exact for a plane wave and a good approximation provided that the pulses are not strongly focused.

10 particles per cell are used with 8 cells per beat wavelength. The seed (pump) characteristics are central wavelength, ${\lambda }_{c}=1.147(1.053)$ μm, full bandwidth, Δ*λ* = 15(≤2) nm, pulse duration (intensity at FWHM), $\tau =1(10)$ ps, linear optical chirp, *α* = 3.87% (0.154%), beam waist, *w* = 250 (50) μm. The initial seed amplitude is *a*_1_ = 1.13 × 10^−6^, and 3 different pump amplitudes are used, *a*_0_ = 8.98 × 10^−3^, 2.84 × 10^−2^ and 8.98 × 10^−2^ representing powers of 1 × 10^14^, 1 × 10^15^ and 1 × 10^16^ W cm^−2^, respectively. The plasma density is chosen to be 6.38 × 10^18^, and the plasma temperature is set to 10 eV, 7 eV and 3 eV for pump powers 1 × 10^14^, 1 × 10^15^ and 1 × 10^16^ W cm^−2^, respectively. Resonant conditions are perfectly matched for the lowest pump amplitude, corresponding to the experimental optimization. The plasma density profile consists of a linear up/down ramp of 150 μm and a flat top of 1.5 mm. An angle of 175° is used between the seed and pump. Leap^49^. The laser pulses are modeled by two counterpropagating envelope fields, which couple to an electrostatic PIC solver through the ponderomotive force. Particle positions are used to calculate the refractive properties of the plasma, which are then used to calculate dispersion and the coupling between the two pulses to update the laser fields. The relatively low resolution required to model the laser envelopes means that the model has a significantly lower computational overhead than conventional electromagnetic PIC codes. The low resolution has the additional advantage that the optical characteristics are based on integrals of the electron distribution over a relatively large volume, making simulations less susceptible to instabilities growing from noise, such as N-RS.

### 1-dimensional simulations

1D-numerical simulations have been performed using the fully explicit, fully relativistic, PIC code OSIRIS^47^ in a static window. The number of particles per cell is 640 with 50 cells per laser wavelength. Plasma density and pump beam parameters are identical to the ones used for the 2D-simulations, except for the fact that the pump beam is monochromatic. The plasma density profile consists of a linear up/down ramp of 200 μm and a flat top of 1.1 mm.

### Data Availability

Data associated with research published in this paper is accessible at http://dx.doi.org/10.15129/2fd625bf-2d54-4514-a664-04876145f6a8.

## Electronic supplementary material

Supplementary Information
