# Supplementary material for: An ultra-high gain and efficient amplifier based on Raman amplification in plasma
Source: Sci Rep. 2017 May 25;7:2399. doi: 10.1038/s41598-017-01783-4 (PMC5445100; doi:10.1038/s41598-017-01783-4)
Supplement: Supplementary file 1 — Supplementary Information [file 41598_2017_1783_MOESM1_ESM.pdf]

An ultra-high gain and efficient amplifier based on Raman amplification in plasma

G. Vieux,[1,2,a] S. Cipiccia,[1,b] D. W. Grant,[1] N. Lemos,[3,c] P. Grant,[1] C. Ciocarlan,[1,4] B. Ersfeld,[1] M. S. Hur,[5] P. Lepipas,[1] G. G. Manahan,[1] G. Raj,[1,d] D. Reboredo Gil,[1] A. Subiel,[1,e] G. H. Welsh,[1] S. M. Wiggins,[1] S. R. Yoffe,[1] J. P. Farmer,[6] C. Aniculaesei,[1,f] E. Brunetti,[1] X. Yang,[1,g] R. Heathcote,[7] G. Nersisyan,[8] C. L. S. Lewis,[8] A. Pukhov,[6] J. M. Dias,[3] and D. A. Jaroszynski[1,h]

[1]*Scottish Universities Physics Alliance and University of Strathclyde, Glasgow G4 0NG, United Kingdom*

[2]*Institute of Physics of the ASCR, ELI-Beamlines, Na Slovance 2, 182 21 Prague, Czech Republic*

[3]*GoLP/Instituto de Plasmas e Fusão Nuclear, Instituto Superior Técnico, Universidade de Lisboa, Lisbon, Portugal*

[4]*IFIN-HH, National Institute for Physics and Nuclear Engineering, Bucharest, Romania*

[5]*UNIST, Banyeon-ri 100. Ulju-gun, Ulsan 689-798, South Korea*

[6]*Theoretische Physik I, Heinrich Heine Universität, 40225 Düsseldorf, Germany*

[7]*Central Laser Facility, Rutherford Appleton Laboratory, Didcot OX11 0QX, United Kingdom*

[8]*Centre for Plasma Physics, School of Mathematics and Physics, Queens University Belfast, Belfast, BT7 1NN, United Kingdom*

(Dated: May 3, 2017)

EXPERIMENTAL SETUP

In the following we expand on the description of the experimental setup given in the main text. A simplified schematic of the experimental layout is presented in Supplementary Figure 1. Only the main diagnostic elements are shown.

The pump beam. The pump consists of a 10-ps long, 1.053-µm central wavelength laser pulse with an energy up to 150 J before compression ($\sim 70\%$ on target, 25-30% within the beam waist). It is focused into a 3 mm long, hydrogen gas jet by a $f/21$ lens, giving a $\sim 50\,\mu$m focal waist.

The seed beam. During the first experimental campaign, the seed was produced by frequency down-shifting a 150-mJ, 1-ps long, 1.053-µm laser pulse in a KGW Raman crystal. A telescope is used to reduce the beam size by a factor of ~ 10 before it propagates through the Raman shifter crystal that has dimensions $20 \times 20 \times 10$ mm^3 [1]. Less than 10% of the energy is down-shifted to 1.147 µm. Two notch filters are then used to block the remaining 1.053 µm radiation. Due to the poor optical quality of the seed, it is passed through a spatial filter consisting of a 600 µm pin-hole placed in the focal plane of a 1:1 telescope. The seed is focused into the gas jet by a $f/40$ plano-convex lens. Deterioration of the beam quality due to nonlinear effects results in a focal waist of 1.5 mm. As a consequence the pump beam is contained inside the seed beam in the plasma region, which facilitates complete spatial overlap. The energy of the seed is around 130 nJ, of which only 220($\pm$60) pJ interacts with the pump main focal spot. The seed spectrum has a Lorentzian profile with a FWHM of 15 nm.

Interaction geometry and diagnostic elements. The pump and seed are nearly counter-propagating, with a 175° angle between the two beams, to avoid feedback into the laser chain. A 3″ wedge is used to collect 1% of the light propagating along the seed axis after interaction and transport it to the diagnostics table. A calorimeter is set up behind the wedge to directly measure the energy. The diagnostics system includes a 14-bit infra-red CCD camera (Xenics), to image the beam at the gas jet, and a Shamrock spectrometer with a 150 lines/mm grating coupled to a 16-bit, deep depletion CCD camera (Andor). In addition to neutral density filters for attenuating the signal energy, notch filters (1064[$\pm$20] nm) are used in front of all the diagnostic components to ensure that radiation at 1.053 µm is absent from the measurements. The diagnostic system for the pump includes an imaging system comprising

a 14-bit CCD camera (AVT Stingray) with a $\times 10$ magnification microscope objective for beam alignment and observation, a 16-bit Ocean Optics spectrometer, and a calorimeter. During the second campaign, the Raman signal scattered exactly in the backward direction with respect to the pump is monitored. Light transmitted through the final pump mirror (before the gas jet) is transported to a calorimeter for energy measurements and focused into a Shamrock imaging spectrometer coupled to a 16-bit, deep depletion CCD camera (Andor) for spectral measurements.

The plasma. To achieve resonance between pump and seed, a plasma density of $6 - 7 \times 10^{18}$ cm^{-3} is required. To this end, the laser beams are focussed 1.25 mm above the gas nozzle with the jet operating at a backing pressure of 12 bars. Hydrogen gas has been used because of its low ionisation threshold, which restricts the pump intensities to above 10^{14} W cm^{-2}.

DISCUSSION ON LIMITATIONS OF NUMERICAL CODES

Simulations of Raman amplification are challenging, especially in regimes where noise is significant. The excited plasma wave has a wavelength half that of the laser and becomes anharmonic at high amplitudes, which requires very high resolution simulations. The presence of wavebreaking and kinetic effects make three-wave models (see for example ref. [2]) unsuitable for this regime, and constraints on the computational overhead limit Vlasov simulations to one dimension[3], therefore PIC simulations are used. However, the number of macro-particles used in simulations is much smaller that the actual number of electrons, which results in a substantial noise component acting as a distributed seed of the amplification.

`cplPIC` is based on ref. [4] and uses a fully-electromagnetic treatment of the laser pulses, and incorporates a moving-window geometry, which allows two-dimensional simulations to be carried out in a non-collinear geometry. However, numerical noise can lead to unphysical results in a moving frame because the perturbation of the plasma by the pump beam before arrival of the seed is not accounted for. In addition, amplification occurs at the leading edge of the window, which results in a short pulse because of strong wavebreaking occurring over the rest of the window, as illustrated in Supplementary Figure 2.

The `Leap` model[5] uses an envelope treatment of the laser pulses and approximates the

low-phase-velocity plasma wave as an electrostatic wave, which allows a significant reduction in computational overhead. However, the envelope treatment limits the interaction to a counterpropagating geometry.

Finally, the optimised parallelisation scaling of the `OSIRIS` code[6] makes it the preferred choice for high-resolution one-dimensional simulations in a fixed window, which avoids the problems of a moving window as described above. However, the noise signal is artificially enhanced by the small number of macro-particles.

Note that ionization and IB heating are not modeled because of the large computational overhead.

ADDITIONAL SIMULATION RESULTS

Multi-dimensional effects. As mentioned above, while simulations fail to give good quantitative agreement with measurements, they can qualitatively illuminate the physical processes that are important. Results from `cplPIC`, presented in Supplementary Figure 2, highlight two possible deleterious multi-dimensional effects: filamentation and Raman side-scattering.

The spatial profiles obtained from the simulations show clear structure in the form of filaments, each of which contain different spectral components. These are already evident for the lowest pump intensity ($1 \times 10^{14}\,\mathrm{W\,cm^{-2}}$), and while they may be over-estimated, it clearly indicates that the pump intensity should be kept to a moderate value.

At higher pump intensities, Raman side scattering acts to broaden the Raman spectrum, in particular towards longer wavelengths because of the phase-matching conditions. The simulated spectral bandwidths are larger than those measured, which may be due to the lack of detailed knowledge of the interaction region geometry.

Damping of the plasma wave. To obtain qualitative agreement between the measured and calculated seed energies, a fixed damping term for the plasma wave has been incorporated into the `Leap` code. Results are presented in Supplementary Figure 3. 3 values, $\nu/\omega_p = 0.002$, 0.02 and 0.5 have been used, respectively, representing realistic plasma temperatures, 0–80 eV. It is observed that damping of the plasma wave can strongly affect the amplification process. Initial results show that damping delays the onset of wavebreaking and leads to an exponential growth of the seed energy for lower pump intensities. However,

for a more realistic comparison a damping factor depending on the pump energy is required, which is consistent with the change in plasma temperature due to IB damping of the pump. A self-consistent model calculating the evolution of the plasma temperature due to the pump has not yet been available at this stage, but is work in progress.

Simulations with a seed of moderate intensity. From the simulation results presented in the main body of the paper, it appears that wavebreaking from early pump backscattering from noise could constitute a serious limitation to efficient amplification of the seed. To assess this deleterious effect, 1D `OSIRIS` simulations have been undertaken for seed beams with intensities just below 10^{14} W cm^{-2} for two different pump intensities, 10^{14} W cm^{-2} (Supplementary Figures 4a-d) and 10^{15} W cm^{-2} (Supplementary Figures 4e-h), respectively. The simulation parameters are identical to the ones used in the previous 1D simulations, except that the number of particles per cell is 64. The seed duration is 1 ps, which matches the experimental conditions.

The run conducted with the lowest pump intensity results in an amplified seed reaching a peak intensity of 6×10^{14} W cm^{-2}, with a pulse duration compressed down to 275 fs as illustrated in Supplementary Figure 4d. The energy transfer efficiency is 21% with 68% of the energy contained within the main peak and 12% of the energy contained in a pedestal in front of this peak. This scattered energy is the result of SRBS from noise prior to the seed arrival. After propagating through $\sim 1/3$ of the plasma length, wavebreaking develops behind the leading edge of the seed, giving rise to pulse compression because no amplification is possible at the back of the pulse (see Supplementary Figure 4b). Scattering from noise in front of the seed is not sufficiently strong to disrupt the plasma significantly. Only after propagating through $\sim 2/3$ of the plasma does the seed encounter plasma electrons with their coherence destroyed by wavebreaking from amplification of noise (see Supplementary Figure 4c). In summary, use of a moderate seed and pump intensity leads to significant pulse amplification and compression, where SRBS from noise is kept under control as is indicated by our experimental results.

In contrast, the run with higher pump intensities leads to the scattering of a strongly modulated signal field comprising a train of ultrashort pulses, which is similar to that obtained from amplification of noise, as shown in Supplementary Figure 4h. In this case, the energy transfer efficiency is 7.5%, which is identical to that obtained from SRBS from noise. The only difference is that a higher peak intensity of 1.8×10^{15} W cm^{-2} is reached. It is

observed from Supplementary Figure 4f that wavebreaking develops very early, before the seed reaches the plasma. This has two consequences: (i) a large signal from noise will be backscattered and amplified extremely quickly; (ii) the seed propagates in a highly perturbed plasma medium in which phase mixing prevents efficient amplification, except at randomly distributed regions (see Supplementary Figure 4g).

If one assumes a moderate initial seed intensity, it is clearly advantageous to also use a moderate pump intensity for cases where no control over the plasma or pump parameters is considered. Even without optimising the seed profile and duration it seems possible to obtain amplification and compression with a good energy transfer efficiency. Further optimisation, and control of the plasma density profile, seed and pump characteristics are certain to increase the efficiency further.

[a] g.vieux@strath.ac.uk

[b] Current address: Diamond Light Source, Harwell Science and Innovation Campus, Fermi Ave, Didcot OX11 0DE, UK

[c] Current address: Lawrence Livermore National laboratory, NIF and photon Sciences, 7000, East avenue, Livermore, CA 94550, USA

[d] Current address: Centre de Physique Théorique, École Polytechnique, 91128 Palaiseau cedex, France

[e] Current address: National Physical Laboratory, Medical Radiation Science, Hampton Road, Teddington, Middlesex, TW11 0LW, UK

[f] Current address: Center for Relativistic Laser Science, Institute for Basic Science, Gwangju 61005, Republic of Korea

[g] Current address: Department of Physics, Capital Normal University, Key Lab of Terahertz Optoelectronics, Ministry of Education, and Beijing Advanced Innovation Center for Imaging Technology, Beijing 100048, China

[h] d.a.jaroszynski@strath.ac.uk

[1] Major, A., Aitchison, J. S., Smith, P. W. E., Langford, N. & Ferguson, A. I. Efficient Raman shifting of high-energy picosecond pulses into the eye-safe 1.5-μm spectral region by use of a $KGd(WO4)_2$ crystal. *Opt. Lett.* **30**, 421 (2005).

[2] Malkin, V. M., Shvets, G. & Fisch, N. J. Fast Compression of Laser Beams to Highly Overcritical Powers. *Phys. Rev. Lett.* **82**, 4448 (1999).

[3] Toroker, Z., Malkin, V. M. & Fisch, N. J. Backward Raman amplification in the Langmuir wavebreaking regime. *Phys. Plasmas* **21**, 113110 (2014).

[4] Hur, M. S. & Wurtele, J. S. Two-dimensional simulations of the amplification and focusing of intense laser pulses in the kinetic regime of Raman backward amplification in plasmas. *Comp. Phys. Comm.* **180**, 651–655 (2009).

[5] Farmer, J. P. & Pukhov, A. Fast multidimensional model for the simulation of Raman amplification in plasma. *Phys. Rev. E* **88**, 063104 (2013).

[6] Fonseca, R. A. *et al.* *Computational Science — ICCS 2002: International Conference Amsterdam, The Netherlands, April 21–24, 2002 Proceedings, Part III*, chap. OSIRIS: A Three-Dimensional, Fully Relativistic Particle in Cell Code for Modeling Plasma Based Accelerators, 342–351 (Springer Berlin Heidelberg, Berlin, Heidelberg, 2002).

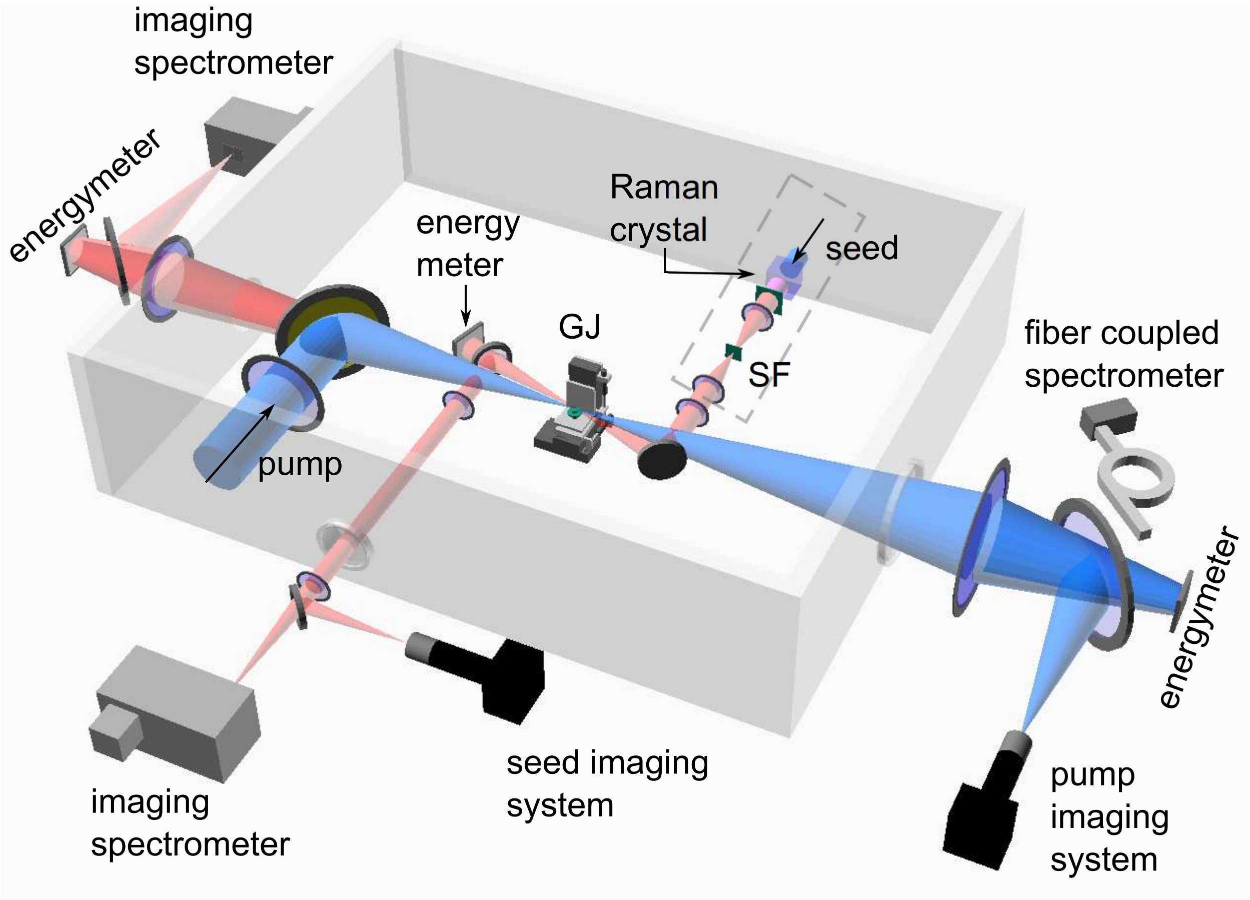

Supplementary Figure 1. **Simplified experimental setup**. The schematic presents the interaction region and the main diagnostic elements. GJ and SF refer to gas jet and spatial filter, respectively. The dashed-line rectangle outlines the seed production setup used during the first experimental run. The diagnostic elements used to measure the Raman signal directly backscattered on the pump axis were only installed for the second run.

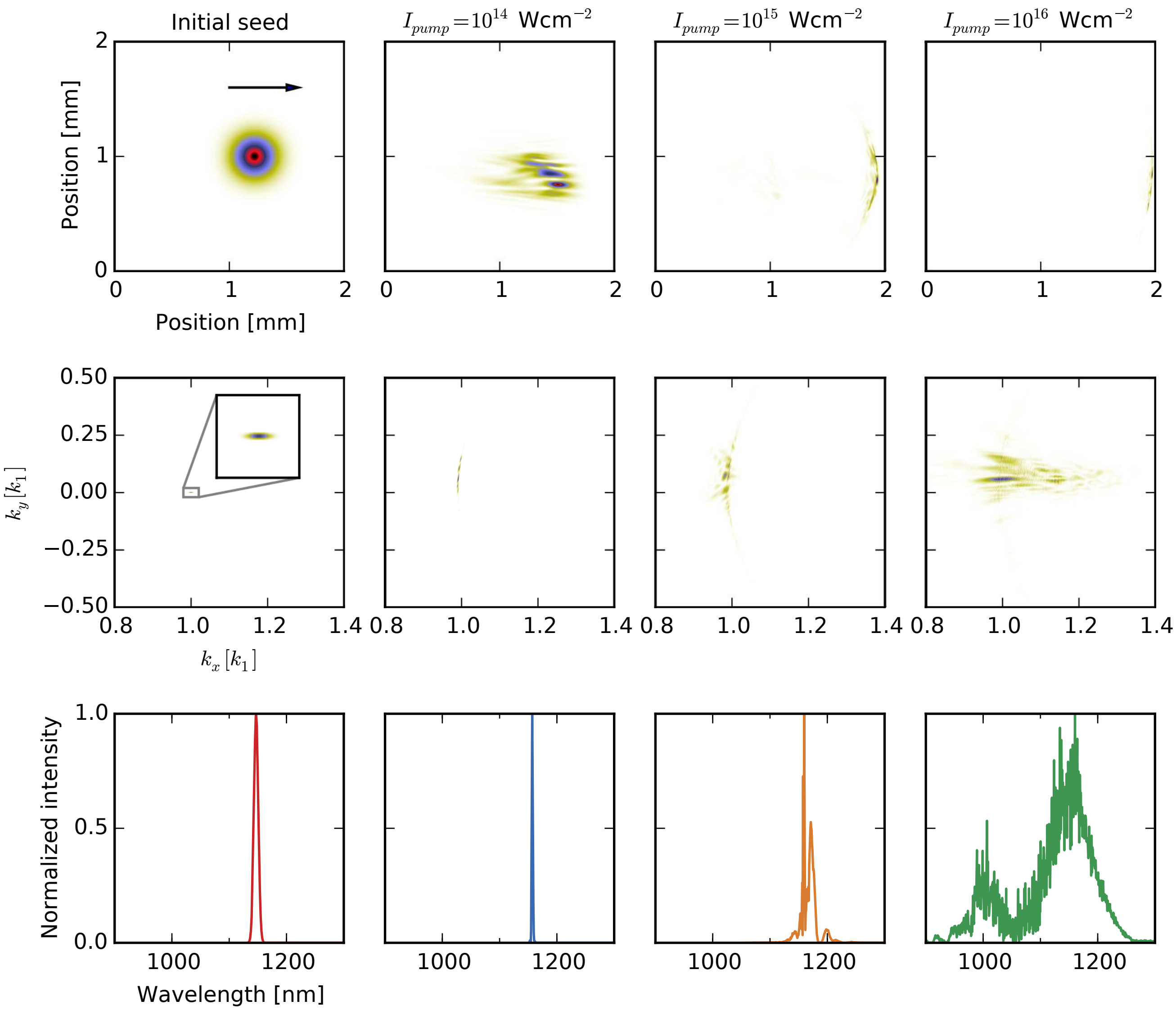

Supplementary Figure 2. **2-dimensional simulation results.** Initial and amplified seed characteristics for 3 different pump intensities: 1×10^{14}, 1×10^{15} and 1×10^{16} W cm^{-2}. Top: seed spatial intensity. Middle: corresponding spectral intensity. Bottom: corresponding horizontal lineout taken at the center of the spectrum.

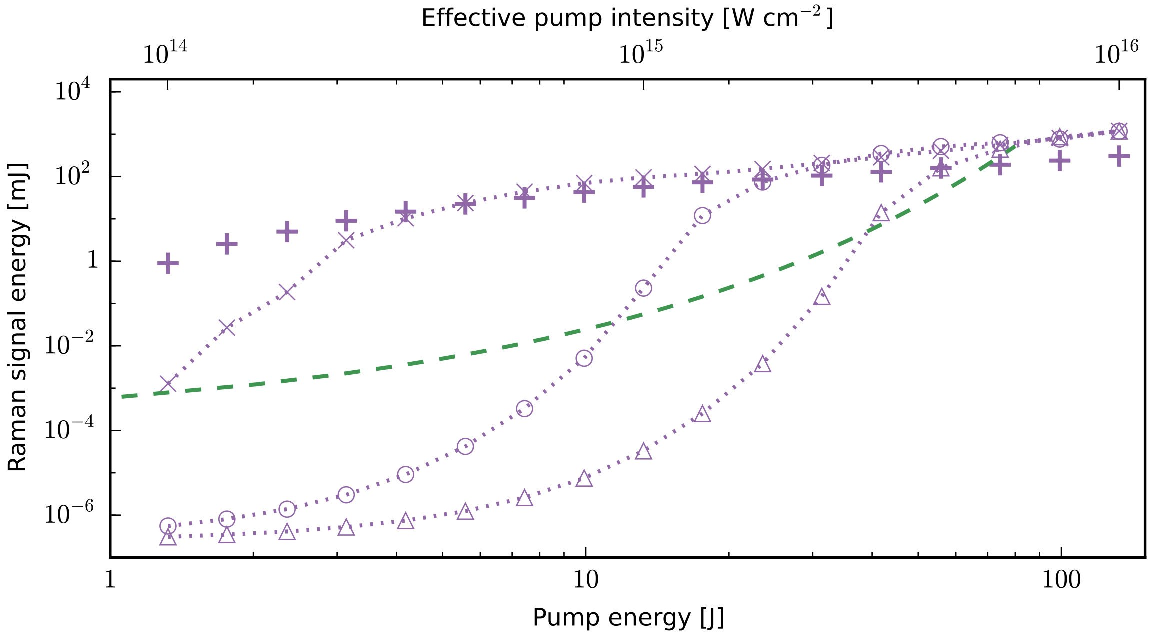

Supplementary Figure 3. **Numerically calculated Raman signal energy from `Leap`.** Energies are obtained for: (+) an undamped plasma wave; (x) a damping factor $\nu/\omega_p = 0.002$; (o) a damping factor $\nu/\omega_p = 0.02$; (triangle) a damping factor $\nu/\omega_p = 0.05$. Damping factors have been chosen to represent realistic plasma temperatures of $1 - 80\,\mathrm{eV}$. For comparison, the best fit to the experimental data is included (dashed line).

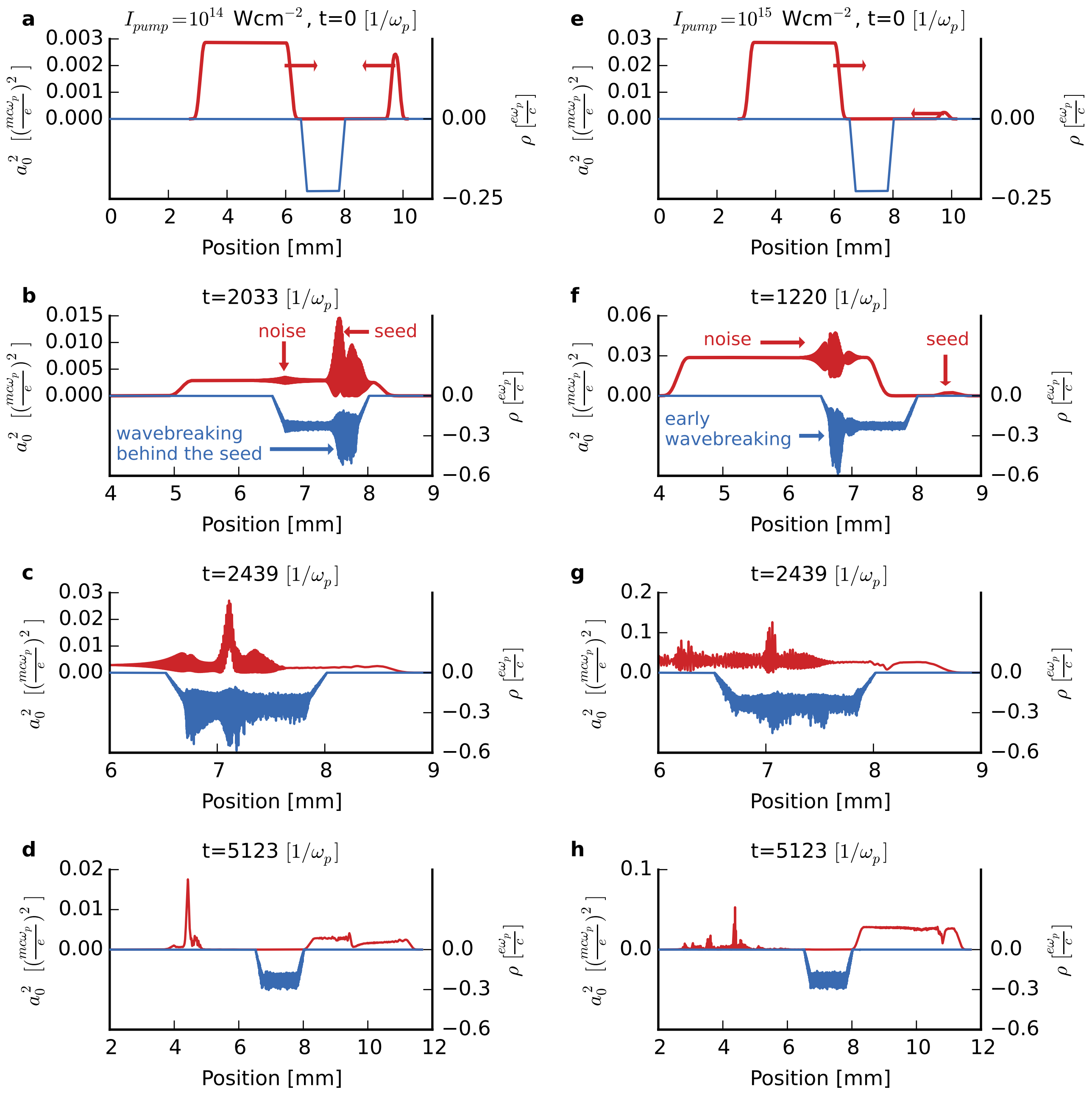

Supplementary Figure 4. **1D simulation results of seed amplification.** Evolution of Raman amplification of a seed pulse for two different pump intensities: 10^{14} W cm^{-2} (**a-d**) and 10^{15} W cm^{-2} (**e-h**). The transverse electric fields (laser pulses) are represented in red, and the plasma densities in blue.
